# Prognostic value of decreased expression of RBM4 in human gastric cancer

**DOI:** 10.1038/srep28222

**Published:** 2016-06-21

**Authors:** Hongmei Yong, Huijun Zhu, Shu Zhang, Wei Zhao, Wei Wang, Chen Chen, Guipeng Ding, Lun Zhu, Ziyuan Zhu, Huaidong Liu, Yongjie Zhang, Jinbo Wen, Xing Kang, Jin Zhu, Zhenqing Feng, Baorui Liu

**Affiliations:** 1 Department of Pathology, Nanjing Medical University, Nanjing 210029, China; 2 Department of Oncology, Huai’an Hospital Affiliated of Xuzhou Medical College and Huai’an Second People’s Hospital, Huai’an 223002, China; 3 Department of Pathology, Affiliated Hospital of Nantong University, Nantong 226019, China; 4 School of Public Health, Nantong University, Nantong 226000, China; 5 Department of Oncology, Second Affiliated Hospital, Nanjing Medical University, Nanjing 210029, China; 6 Department of Epidemiology, School of Public Health, Nanjing Medical University, Nanjing 210029, China; 7 Department of General Surgery, Drum Tower Hospital, Medical School of Nanjing University, Nanjing 210008, China; 8 Key Laboratory of Antibody Technique of Ministry of Health, Nanjing Medical University, Nanjing 210029, China; 9 Huadong Medical Institute of Biotechniques, Nanjing 210029, China; 10 Jiangsu Collaborative Innovation Center For Cancer Personalized Medicine, Nanjing Medical University, Nanjing 210029, China; 11 The Comprehensive Cancer Center, Drum Tower Hospital, Medical School of Nanjing University & Clinical Cancer Institute of Nanjing University, Nanjing 210008 China

## Abstract

RNA-binding motif 4 (RBM4) is a multifunctional protein that participates in regulating alternative splicing and mRNA translation. Its reduced expression has been associated with poor overall survival in lung cancer, breast cancer and ovarian cancer. We assessed RBM4 protein expression levels with immunohistochemistry in tissue microarrays containing malignant gastric cancer tissues and benign tissues from 813 patients. We also examined the expression levels of RBM4 mRNA in twenty-five paired gastric cancer samples and adjacent noncancerous tissues. Both RBM4 protein and mRNA expression levels were significantly lower in gastric cancer tissues compared with the adjacent noncancerous tissues. There was a significant association between reduced RBM4 protein expression and differentiation (*P* < 0.001), lymph node metastasis (*P* = 0.026), TNM state (*P* = 0.014) and distant metastasis (*P* = 0.036). *P*atients with reduced RBM4 expression (*P* < 0.001, CI = 0.315–0.710) and TNM stage III and IV (*P* < 0.001, CI = 4.757–11.166) had a poor overall survival. These findings suggest that RBM4 is a new biomarker in gastric cancer, as the reduced expression of this protein is correlated with poor differentiation, lymph node status and distant metastasis. Further, lower RBM4 expression is an independent prognostic marker for gastric cancer.

Even though its incidence has been decreasing during past decades, Gastric cancer remains as the second leading cause of cancer death worldwide^1^. More than 70% of the gastric cancer cases occur in developing countries and China has the highest incidence^2^. Gastric cancer is the third leading cause of death among cancer patients in China with an age standardized incidence of 22.7 per 100,000^3^. Metastatic spread of tumor cells from the stomach to other sites, such as the peritoneum and liver, is the cause of the high mortality rates in gastric cancer patients^4^. A variety of significant treatments have been used with early-stage gastric cancer patients. However, the overall prognosis of gastric cancer patients at advanced stages is still unfavorable in spite of aggressive treatments^5^,^6^. Therefore, it is very important to identify novel biological markers for gastric cancer that can help us develop an early diagnosis method and target therapy for extending the life of gastric cancer patients.

Initially described in Drosophila, RNA-binding motif 4 (RBM4), which is also known as Lark, is expressed ubiquitously throughout development, with relatively high abundance in heart, brain, skeletal muscle and testis^7^. It is particularly highly expressed in the embryonic nervous system^8^. In humans, the gene is located on chromosome 11q13 and encodes a protein of 364 -amino acids long^9^,^10^. The human gene has 95% homology with the mouse ortholog and 53% homology with the Xenopus homolog. Two RNA recognition motifs (RRMs) and a CCHC-type zinc finger in the N-terminal region have been found in RBM4, whereas a low-complexity region is located in the C-terminal that can interact with other proteins^11^.

RBM4 is a multifunctional protein that participates at least in modulating alternative splicing and mRNA translation^12^,^13^. RBM4 can regulate exon selection and alternative splicing in both *in vivo and in vitro* splicing models^14^,^15^. RBM4 has been shown to regulate translation, under cell stress by interacting directly with argonaute 2^16^, inhibiting cap-dependent translation^13^, mediating an oxygen-regulated translation switch^11^ or activating internal ribosomal entry site-mediated translation^17^. Furthermore, RBM4 is able to selectively associate with specific microRNAs in muscle cells and repress their translation activity by promoting micro-ribonucleoprotein connection with target mRNAs^18^. In addition, overexpression of RBM4 promotes differentiation of pancreas and muscle cells^19^,^20^. Even though RBM4 has been reported to be a tumor suppressor that inhibits lung cancer progression in both cultured cells and in a tumor xenograft model^21^, little is known about RBM4 expression in cancer.

In this study, we sought to explore the role of RBM4 expression in the prognosis of gastric cancer patients. RBM4 expression was assessed by immunohistochemistry and qRT-PCR techniques. The association of RBM4 expression with clinicopathologic characteristics and overall survival (OS) was evaluated as well.

## Results

### Reduced RBM4 mRNA expression in cancer

**Figure 1:**
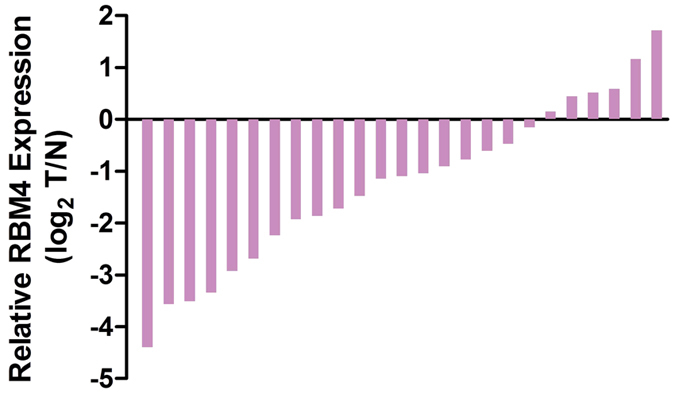
mRNA expression of RBM4 in paired gastric cancer samples and adjacent noncancerous tissue. Quantitative polymerase chain reaction (qRT-PCR) was performed to detect the expression of RBM4 mRNA in twenty five gastric cancer compared with adjacent noncancerous tissues using β-actin, as a normalization control. RBM4 mRNA levels were significantly lower in gastric cancer compared with corresponding noncancerous tissues (*P* < 0.001). Each bar represents one patient on the x-axis.

We measured RBM4 mRNA levels in 25 paired gastric cancer samples and adjacent noncancerous tissues. The results showed that 19 primary gastric cancer samples had substantially reduced RBM4 expression levels on mRNA compared with the paired adjacent noncancerous tissues, with an average downregulation fold of 0.643 (*P* < 0.001) (Fig. 1).

### Clinicopathologic features of patients

Using TMAs, we analyzed RBM4 protein expression in 813 (94.4%) of the 861 samples in the TMA, The remaining samples were lost during antigen retrieval or there were no tumor tissues found in the core. The level of core loss was similar to the loss rates described in previous TMA studies^22^.

Of the 813 benign and malignant gastric tissues analyzed (Table 1, Supplementary dataset 1), there were 29 chronic gastritis, 25 intestinal metaplasia, 24 low-grade intraepithelial neoplasia, 27 high-grade intraepithelial neoplasia, 103 matched adjacent noncancerous and 605 cancer. The baseline characteristics of the 605 gastric cancer were shown in Table 2 (Supplementary dataset 2). There were 458 male and 157 female patients. Their median age was 59.5 years (range, 33–86). The distribution of TNM stage was as follows: 352 patients at stage 0, I and II, 253 at stage III and IV. For the histological type, most patients initially presented as the tubular (528/605) type. For the differentiation status, 37 tumors were well-differentiated, 224 were moderately- differentiated, 278 were poorly- differentiated, and 66 were others (not of the tubular or papillary adenocarcinoma type).

### RBM4 protein expression in benign and malignant gastric tissues by IHC

**Figure 2:**
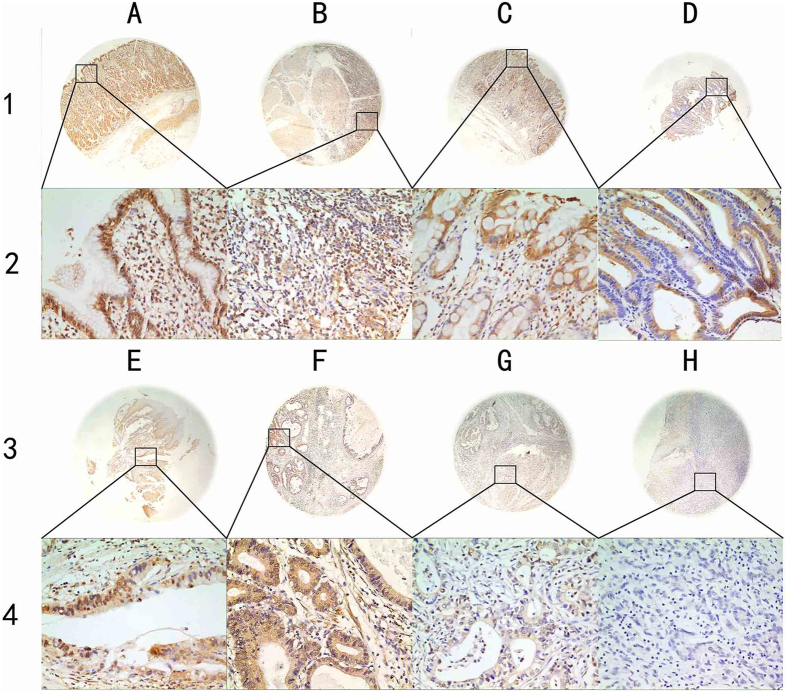
Representation of RBM4 protein expression in gastric benign and malignant tissues on TMA sections. Column A: normal surgical margin of gastric cancer with high RBM4 expression (IHC score, 270); column B: chronic gastritis with high RBM4 expression (IHC score, 140); column C: intestinal metaplasia with high RBM4 expression (IHC score, 160); column D: low-grade intraepithelial neoplasia with high RBM4 expression (IHC score, 110); column E: high-grade intraepithelial neoplasia with low RBM4 expression (IHC score, 90); column F: well differentiated gastric cancer with high RBM4 expression (IHC score, 180); column G: moderately- differentiated gastric cancer with low RBM4 expression (IHC score, 20); column H: poorly differentiated gastric cancer with low RBM4 expression (IHC score, 0). Row 1 and 3 are RBM4 staining with ×40 magnification, and row 2 and 4 are RBM4 staining with ×400 magnification.

RBM4 protein expression mostly presented in the cytoplasm and nucleus, especially in nuclear speckles (Fig. 2). Using the X-tile software program for TMA data analysis (http://www.tissuearray.org/rimmlab), we first identified the significant cutoff point in terms of OS in gastric cancer. We found the appropriate cutoff point to be 100: Score 0–100 was considered no or low expression while 101–300 was considered high expression. For the subsequent analyses, RBM4 protein expression levels were considered either as “Low or no” or “High” using these cutoff values.

High RBM4 protein expression was recorded in 58.6%, 56.0%, 54.2%, 51.9% and 63.1% of the stomach benign tissues in chronic gastritis, intestinal metaplasia, low-grade intraepithelial neoplasia, high-grade intraepithelial neoplasia and adjacent noncancerous tissues, respectively (Table 1). In gastric cancer, high RBM4 protein expression was only 43.8%, significantly lower than in the benign tissues (*P* = 0.006).

### Association of RBM4 expression with clinicopathologic characteristics in gastric cancer

Table 2 shows a summary of the correlations between RBM4 protein expression levels and clinicopathologic variables in the gastric cancer patients. Reduced RBM4 expression was significantly associated with poor differentiation (*P* < 0.001), lymph node metastasis (*P* = 0.026), distant metastasis (*P* = 0.036) and advanced TNM stage (*P* = 0.014). However, we did not find a significant correlation between RBM4 expression with depth of invasion and other clinicopathologic variables, including age, sex and histological type.

### Reduced RBM4 expression correlates with poor OS

**Figure 3:**
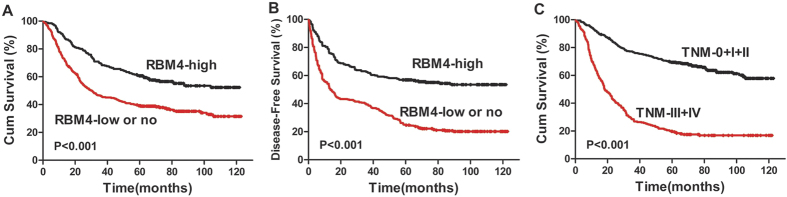
Correlation between RBM4 expression and clinicopathologic parameters and patients survival in human gastric cancer. (**A**) Low or no RBM4 expression correlates with a poorer overall cumulative survival for 605 gastric cancer patients (*P* < 0.001, log-rank test). (**B**) Low or no RBM4 expression was found to be correlated with a poorer disease-free survival for 605 gastric cancer patients (*P* < 0.001, log-rank test). (**C**) TNM III + IV correlates with a poorer overall cumulative survival for 605 gastric cancer patients (*P* < 0.001, log-rank test).

To evaluate the predictive value of RBM4 expression in gastric cancer, Kaplan-Meier survival curves were performed to compare the patients with high RBM4 expression and those with low or no RBM4 expression using overall cumulative survival. Data showed reduced RBM4 expression to be correlated with worse OS (*P* < 0.001, Fig. 3A) and worse disease-free survival (DFS) (*P* < 0.001, Fig. 3B). For OS, the overall cumulative survival rate was 31.4% in the low or no RBM4 expression groups and 52.4% in the high RBM4 expression group. DFS was 20.9% and 54.7%, respectively. We also found poorer OS in TNM stages III and IV compared with that in TNM stages 0, I and II (*P* < 0.001, Fig. 3C).

The univariate and multivariate analyses results for prognostic markers in gastric cancer are shown in Table 3. Reduced RBM4 expression (HR = 0.449, 95% CI = 0.324–0.624; *P* < 0.001) was significantly associated with a shorter survival in univariate analysis, along with other prognostic factors, including age (HR = 0.711, 95% CI = 0.512–0.987; *P* = 0.042), differentiation (HR = 1.607, 95% CI = 1.290–2.002; *P* < 0.001) and TNM stage (HR = 8.415, 95% CI = 5.691–12.444; *P* < 0.001). In multivariate analysis, both reduced RBM4 expression (HR = 0.470, 95% CI = 0.315–0.710; *P* < 0.001) and TNM stage (HR = 7.288, 95% CI = 4.757–11.166; *P* < 0.001) worsened the prognosis independently (Table 3).

## Discussion

The two isoforms of RBM4 that have been reported in mammals (i.e.,—RBM4a and RBM4b) have a very similar structure and sequence. Interestingly, the entire RBM4a gene is situated within intron 2 of RBM4b^19^. Since RBM4a is the only isoform that has been studied, the present review will only refer to this isoform. Not much is known about the role of RBM4 in cancer. To date, only two studies have investigated the potential role of RBM4 in tumors: Lin *et al.* found that the SRPK1-RMB4 network may contribute to tumorigenesis through altered sensitivity to apoptotic signals in breast cancer cells^23^. Wang *et al.* have shown that only in lung, breast and ovarian cancer, RBM4 expression is decreased dramatically in cancer patients and a reduced RBM4 level is correlated with poor survival^21^. The function of RBM4 in gastric cancer has not been clearly studied.

In this study, we first found that RBM4 staining was localized in the nucleus and cytoplasm, and this observation was in line with previous findings^14^. According to immunohistochemistry and qRT-PCR analyses, the expression of RBM4 protein and mRNA is downregulated in gastric cancer relative to levels in human chronic gastritis, intestinal metaplasia, low-grade intraepithelial neoplasia, high-grade intraepithelial neoplasia and adjacent noncancerous tissues. These results were similar to Wang *et al.*^21^, indicating that inactivation of RBM4 may play an important role in tumorigenesis of gastric cancer. However, our study showed that RBM4 protein expression levels were generally higher (43.8%) than Wang’s study (19.0%) in cancers, and the reasons may be as follows: 1) different secondary antibodies were used: Abcam company (Wang study) vs Dako company (our study); 2) different research objects: ovarian cancer, non small cell lung cancer and pancreatic cancer (Wang study) vs gastric cancer (our study), as the expression of RBM4 protein may not be the same in different tumors.

Subsequently, we studied the correlation between RBM4 protein expression levels and clinicopathologic variables in gastric cancer patients. We found that reduced RBM4 protein expression was associated with lymph node metastasis, TNM stage and distant metastasis. There was an especially obvious correlation with differentiation (*P* < 0.001). RBM4 protein expression is only in 34.5% of poorly-differentiated cancers, while we found it in 54.5% of moderately-differentiated and 67.6% of well-differentiated cancers. RBM4 promotes myoblast differentiation by selectively binding to muscle-specific miR-1 and miR-206^19^ and elevates pancreas cell differentiation via alternative splicing regulation^20^. However, it is not known how RBM4 mediates gastric cancer cell differentiation. A prospective study is needed to clarify this. Although this study did not indicate a significant correlation between RBM4 expression and histological type, RBM4 protein expression levels were found to be only 17.4% in signet ring cell carcinoma (SRCC), much lower than in any other form of gastric cancer. SRCC is a poorly differentiated cancer, which has been thought to have a worse prognosis in advanced gastric cancer^24^,^25^,^26^, For this reason, identifying the various types and stages of gastric cancer could potentially increase curative effect of targeted therapies. The current study is limited by its small sample size and retrospective design. Future studies with larger samples of SRCC could help clarify the role of RBM4 in gastric cancer.

In addition, we showed that lower expression levels of RBM4 were closely associated with reduced OS and DFS in patients with gastric cancers. Also, RBM4 was an independent gastric cancer prognostic factor according to multivariate Cox regression analysis. These results are consistent with what was found by Wang *et al.*^21^, implying RBM4 may be used as a new therapeutic target for gastric cancer. Of course, it is not only a single gene that is involved in each step of the metastasis process in the clinic of gastric cancer. It has been reported previously that some known factors are associated with RBM4 in cell and mouse models, including SR protein kinase 1(SRPK1)^23^, Bcl-x^21^, CD44^27^ and SRSF1^21^. Future studies are needed to directly compare these factors of protein expression with RMB4 protein expression by IHC in gastric cancer and their association with OS.

It has been reported that RBM4 affects cell growth by controlling both splicing and translation. Splicing dysregulation has been recently shown to be a major molecular hallmark of human cancer^28^,^29^. Our study shows that RBM4 expression is reduced in gastric cancer and RBM4 may serve as a tumor suppressor. Oncogene activation and tumor suppressor gene inactivation are studied most often in tumorigenesis, cancer development and cancer progression^30^. Tumor suppressor genes most often activate antiproliferative and pro-apoptotic pathways and they, therefore, protect cells from advancing to cancer. However, it is not known yet how RBM4 inhibits tumor growth in gastric cancer. Wang *et al.* showed that RBM4 shifts splicing of Bcl-x to suppress cancer progression by controlling the balance between pro- and anti-apoptotic pathways, and RBM4 counteracts SRSF1 to inhibit tumor progression by mediating the activation of the mTORC1 pathway^21^. Further experiments are needed to investigate whether RBM4 interacts directly with Bcl-x and SRSF1 or binds to other splicing factors to suppress tumor progression *in vitro* and in mouse models of gastric cancer.

In conclusion, our study found that RBM4 is a new biomarker in gastric cancer. The reduced expression of RBM4 is correlated with poor differentiation, lymph node status and distant metastasis and is an independent prognostic marker for poor outcome in patient with gastric cancer.

## Materials and Methods

### Patients and tissue specimens

Stomach tissue samples (n = 861) that were formalin-fixed paraffin-embedded (FFPE) and processed in tissue microarray (TMA) were obtained from the Department of Pathology, Affiliated Hospital of Nantong University. The samples included 33 intestinal metaplasia, 27 chronic gastritis, 29 low-grade intraepithelial neoplasia, 31 high-grade intraepithelial neoplasia, 621 gastric cancer tissues and 120 matched adjacent noncancerous tissues. Clinical data included age, sex, histological type, differentiation grade and TNM stage *et al.* Clinical follow-up results were available from 741 patients (range, 2 to 11 years) and at the last follow-up date patients who were alive were censored from the analysis. The date of surgery to death or last follow-up served as the value for OS and the period from surgery to recurrence was defined as the DFS value. No cancer patients received radiation therapy, chemotherapy or immunotherapy before surgery. To perform qRT-PCR test for mRNA expression level, an additional twenty-five freshly gastric cancer tissues and matching adjacent noncancerous tissues obtained from Huai’an Second People’s Hospital, Zhang Jia Gang Ao Yang Hospital, Second Affiliated Hospital of Nanjing Medical University, Drum Tower Hospital of Nanjing Medical University and the First Affiliated Hospital of Nanjing Medical University were also included in this study. This study was approved by the Human Research Ethics Committee of the abovenamed hospitals and carried out in accordance with the approved guidelines. All patients provided written informed consent for their stomach tissue samples to be used for research.

### TMA construction and immunohistochemistry analysis (IHC)

A total 13 gastric TMAs were manufactured by Shanghai Outdo Biotech (China). Core tissue biopsies that were 2 mm in diameter were obtained from ~70 individual FFPE blocks. They were sequentially arranged in one prepared blank paraffin block. To generate TMA slides, four-micron thick sections were cut and mounted on super frost-charged glass microscope slides.

IHC was carried out as previously reported^31^. In brief, we deparaffinized and rehydrated the tissue sections with graded alcohol. The tissue sections were boiled with 0.01 M citrate buffer (pH 6.0) to achieve antigen retrieval. After pre-incubation with 10% FCS (fetal calf serum) for 20 min, the TMA slide was incubated with rabbit anti-human RBM4 antibody (polyclonal, dilution 1:100, Proteintech, Chicago, IL) and subsequently with Envision HRP-congjugated goat anti-rabbit-IgG antibody (monoclonal, dilution 1:100, Dako). A sample in which diluted PBS replaced primary antibody during incubation served as a negative control. All slides were performed at the same time under the same conditions.

RBM4 expression was evaluated by the percentage of cells and the staining intensity with the semi-quantitative H-score method^32^. To evaluate the staining intensity four different groups were defined: 0 = no staining, 1+ = weak staining, 2+ = moderate staining, or 3+ = intense staining. To obtain a final staining score, the intensity score was multiplied by the percentage of cells stained at the respective intensity. Thus the staining score had a minimum value of 0 (no staining) and a maximum of 300 (100% of cells with 3+ staining intensity). Immunostained sections were evaluated by two experienced pathologists under blinded experimental conditions.

### Quantitative real-time polymerase chain reaction (qRT-PCR)

Trizol reagent (Invitrogen, Carlsbad, CA, USA) was used to extract total RNA. RNA quantity and quality were checked by agarose gel electrophoresis and spectrophotometry. Total RNA was reverse transcribed to cDNA using a PrimeScriptTM RT reagent kit (Takara, Glen Burnie, MD). An ABI PRISM 7500HT Sequence Detection System (Applied Biosystems, Foster City, CA, USA) was used for the qPT-PCR reactions. PCR primers were designed with Primer Express Software and 96-well plates were used. Following primers were used for RBM4: forward, 5′-GCAGACTTGACCGAGCAATA-3′ and reverse, 5′- TCCGTACGCGTTGTTGTAAT-3′ (Synthesized by Genescript. Nanjing, China). Human β-actin was used as a control: forward, 5′- TGGAGAAAATCTGGCACCAC-3′ and reverse, 5′- GAGGCGTACAGGGATAGCAC-3′ (Genescript). The reaction system (20 μL) contained 2 μL of cDNA template; each primer, 20 nmol/L and 10 μL of 2 × SYBR Green PCR Master Mixtures (Applied Biosystems). qRT-PCR conditions were as follows: after an initial annealing temperature of 50 °C for 2 min to allow AmpErase-UNG activity and 10 min at 95 °C, the samples were cycled 40 times at 95 °C for 15 sec and 56 °C for 1 min. The Ct-value for each sample was calculated with the ΔΔCt method^33^,^34^,^35^ and results were expressed as 2-^ΔΔCt^. clinicopathological parameters of the 25 cancer patients and primitive Ct-value of qRT-PCR are shown in Supplementary dataset 3 and 4.

### Statistical analysis

SPSS 20.0 statistical software package (SPSS Inc, Chicago, IL) was used for all statistical analyses. Continuous RBM4 expression data from IHC were initially converted into dichotic data (low or no vs high) using specific cutoff values, The cutoff values were selected to be significant regarding OS using the X-tile software program for TMA data analysis (The Rimm Lab at Yale University; http://www.tissuearray.org/rimmlab)^35^,^36^,^37^. To assess the relationship between RBM4 expression and clinicopathological parameters, we used χ^2^ test. Survival curves were made with the Kaplan–Meier method. The significance of differences between the curves was analyzed with a log-rank test. The Cox proportional hazards regression model was used to perform univariate and multivariate analyses. For qRT-PCR, paired-samples *t* test was used. Levels of statistical significance were set at *P* < 0.05.

## Additional Information

**How to cite this article**: Yong, H. *et al.* Prognostic value of decreased expression of RBM4 in human gastric cancer. *Sci. Rep.* **6**, 28222; doi: 10.1038/srep28222 (2016).

## Supplementary Material

Supplementary Dataset 1

Supplementary Dataset 2

Supplementary Dataset 3

Supplementary Dataset 4

## Figures and Tables

**Table 1:** RBM4 expression in gastric benign and malignant tissues.

Characteristics	n	RBM4+	Pearson *X*^2^	*P-value*
			16.151	0.006*
Chronic gastritis	29	17(58.6%)		
Intestinal metaplasia	25	14(56%)		
Low-grade intraepithelial neoplasia	24	13(54.2%)		
High-grade intraepithelial neoplasia	27	14(51.9%)		
Cancer	605	265(43.8%)		
Matched adjacent noncancerous	103	65(63.1%)		

RBM4+ represents high RBM4 expression. *P < 0.05.

**Table 2:** Association of reduced RBM4 expression with clinicopathological characteristics in gastric cancer patients.

Characteristics:	n	RBM4+	Pearson*X*^2^	*P-value*
Total	605	265(43.8%)		
Gender			1.490	0.222
Male	458	207(45.2%)		
Female	147	58(39.5%)		
Age			0.635	0.425
<60	367	156(42.5%)		
≥60	238	109(45.8%)		
Histological type			7.382	0.117
Tubular	528	237(44.9%)		
Mucinous	9	4(44.4%)		
Mixed^a^	34	14(41.2%)		
Signet ring cell	23	4(17.4%)		
Others^b^	11	6(54.5%)		
Differentiation			31.477	<0.001*
Well	37	25(67.6%)		
Moderate	224	122(54.5%)		
Poor	278	96(34.5%)		
Others^c^	66	22(33.3%)		
TNM stage			6.060	0.014*
0+I+II	352	169(48.0%)		
III+IV	253	96(37.9%)		
Depth of invasion			2.695	0.101
Tis+ T1+T2	220	106(48.2%)		
T3+T4	385	159(41.3%)		
Lymph node metastasis			9.272	0.026*
N0	246	123(50.0%)		
N1	107	47(43.9%)		
N2	122	51(41.8%)		
N3	130	44(33.8%)		
Distant metastasis			4.392	0.036*
M0	560	252(45.0%)		
M1	45	13(28.9%)		

^*^P < 0.05.

^a^Mixed: Tubular and mucinous.

^b^others: Papillary adenocarcinoma, 6 cases; Adeno squamous carcinoma, 2 cases; Squamous cell carcinoma, 3 cases; Neuroendoccrine carcinoma, 1 case.

^c^others: other than Tubular and Papillary adenocarcinoma.

**Table 3:** Univariate and multivariate analysis of prognostic markers for overall survival in gastric cancer.

	Univariate analysis			Multivariate analysis		
	HR	*P*-*value*	95% CI	HR	*P*-*value*	95% CI
RBM4 expression	0.449	<0.001*	0.324 0.624	0.470	<0.001*	0.315 0.701
High vs Low or no						
Age (years)	2.012	0.005*	0.512 0.987	0.868	0.330	0.546 1.225
≤60 vs >60						
Gender	1.025	0.923	0.805 1.709			
Male vs Female						
Histological type	1.407	0.020*	0.762 1.095	0.851	0.489	0.538 1.346
Tubular vs Mucinous vs Mixed^a^ vs Signet ring cell vs Others^b^						
Differentiation	0.210	<0.001*	1.549 2.747	1.383	0.053	0.996 1.920
Well vs Middle vs Poor						
TNM stage	8.415	<0.001*	5.691 12.444	7.288	<0.001*	4.757 11.166
0+I+II vs III+IV						
Depth of invasion	5.120	<0.001*	3.576 7.332			
Tis vs T1+T2 vs T3+T4						
Lymph node metastasis	2.320	<0.001*	1.968 2.735			
N0 vs N1 vs N2 vs N3						
Distant metastasis	7.135	<0.001*	2.775 18.346			
M0 vs M1						

HR, Hazard ratio; CI, confidence interval. *P < 0.05.

^a^Mixed: Tubular and mucinous.

^b^others: Papillary adenocarcinoma, 6 cases; Adeno squamous carcinoma, 2 cases; Squamous cell carcinoma, 3 cases; Neuroendoccrine carcinoma, 1 case.
